# Use of Pluronic Surfactants in Gel Formulations of Photosensitive 1,4-Dihydropyridine Derivatives: A Potential Approach in the Treatment of Neuropathic Pain

**DOI:** 10.3390/pharmaceutics13040527

**Published:** 2021-04-10

**Authors:** Giuseppina Ioele, Rita Muzzalupo, Miyase Gözde Gündüz, Michele De Luca, Elisabetta Mazzotta, Fedora Grande, Maria Antonietta Occhiuzzi, Antonio Garofalo, Gaetano Ragno

**Affiliations:** 1 Department of Pharmacy, Health and Nutritional Sciences, University of Calabria, 87036 Rende, Italy; rita.muzzalupo@unical.it (R.M.); michele.deluca@unical.it (M.D.L.); mazzotta-elisabetta@libero.it (E.M.); fedora.grande@unical.it (F.G.); mariaantonietta.occhiuzzi@unical.it (M.A.O.); antonio.garofalo@unical.it (A.G.); gaetano.ragno@unical.it (G.R.); 2 Department of Pharmaceutical Chemistry, Faculty of Pharmacy, Hacettepe University, 06100 Ankara, Turkey; miyasegunduz@yahoo.com

**Keywords:** T-type calcium channel blockers, multivariate curve resolution, drug photostability, Pluronic^®^ surfactants, α-tocopherol

## Abstract

1,4-Dihydropyridines (DHPs) are the most important class of L-type calcium channel blockers that are employed for the treatment of cardiovascular diseases, particularly hypertension. Various modifications on this scaffold lead to the discovery of new DHPs blocking different types of calcium channels. Among them, the T-type calcium channel has recently attracted great interest due to its role in chronic pain conditions. In this study, we selected three newly synthesized DHPs (HM8, HM10 and MD20) with different selectivity profiles to the T-type calcium channel and formulated them in micellar solutions and micellar-in-gel matrices to be tested for potential topical use in the treatment of neuropathic pain. To prevent the well-known sensitivity to light of the DHPs, the studied compounds were entrapped in colloidal aggregates obtained by using edible Pluronic^®^ surfactants and adding α-tocopherol as an antioxidant. All the prepared formulations were exposed to stressing light, according to international rules. Along with the degradation experiments, the concentrations of the parent compounds and by-products were calculated by multivariate curve resolution—alternating least squares (MCR-ALS) applied to the spectral data. The defined formulations proved suitable as light-stable matrices for the DHP compounds, showing an increase in stability for HM8 and MD20 and an almost complete photoprotection for HM10, compared to ethanol solutions and standard gel formulations.

## 1. Introduction

Voltage-gated calcium channels mediate calcium influx into the excitable cells of the brain, heart and smooth and skeletal muscle. They regulate a vast variety of physiological functions, including hormone/neurotransmitter release, gene transcription and muscle contraction in the whole body. Through their abilities to control calcium-dependent processes, calcium channels are regarded as druggable targets for the treatment of cardiovascular and neurological disorders [1]. L-type calcium channel (Ca_v_1.2) is the primary target of 1,4-Dihydropyridines (DHPs), such as nifedipine, amlodipine and isradipine, which are commonly prescribed for controlling hypertension. Subsequently, T-type calcium channels (Ca_v_3) were found to control neuronal excitability and are targeted for the therapeutic intervention into neurophysiologic conditions, principally epilepsy and pain. Among the different T-type calcium channel subtypes, the Ca_v_3.2 isoform regulates pain signals, and thus attracts great interest to bring chronic pain states under control [2].

Within the scope of defining new T-type calcium channel blockers, many compounds of different chemical classes were developed and shown to mediate analgesia [3]. Furthermore, in our previous studies, we synthesized new compounds by introducing a DHP ring into a condensed ring system (hexahydroquinoline) and modifying the alkyl group of the ester side chain, which led to the discovery of novel DHP-based T-type calcium channel blockers with different selectivity profiles over the L-type calcium channel. These derivatives were also demonstrated to be effective at reducing pain signals derived from peripheral inflammation and nerve injury [4,5]. The obtained data suggested that condensed DHPs as T-type calcium channel blockers are novel and promising scaffolds for chronic pain relief.

**Figure 1:**
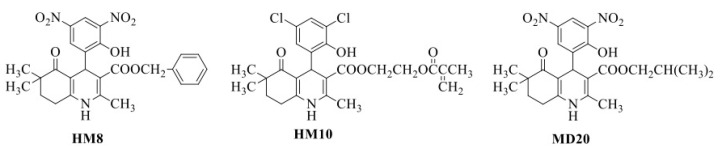
Chemical structures of HM8, HM10 and MD20.

In this study, we selected three new DHPs (HM8, HM10 and MD20) with different selectivity profiles against the T-type over L-type calcium channels, utilizing the whole-cell patch-clamp technique. Their chemical structures are provided in Figure 1. Among them, HM8 and MD20 were selective blockers of the T-type calcium channel (Ca_v_3.2), with a ~60% inhibition over the L-type calcium channel (Ca_v_1.2) with no significant block [6,7]. Besides them, HM10, carrying the Michael acceptor group in the ester side chain, produced almost complete inhibition (>95%) on Ca_v_3.2, exhibiting low selectivity as it caused moderate inhibition (~50%) on Ca_v_1.2 as well [7].

The use of DHPs in therapy has always been difficult due to their known sensitivity to light, which leads to the formation of the pyridine by-product as the major photoproduct. DHP drugs are currently formulated mainly in tablets, due to the high light stability in solid form. Several studies have been carried out for defining liquid formulations, providing a valid photoprotection for these drugs [8,9]. The adoption of dark glass containers is the most used method to protect the few available liquid formulations of DHPs today. Alternatively, matrices based on cyclodextrins, liposomes, niosomes or non-ionic surfactants have been studied as photostabilization systems [10,11,12]. Over the past decade, many efforts have been devoted to the development of drug delivery systems based on the use of nanoscale materials, including polymer micelles and polymer–drug conjugates, in which the drugs can simply be entrapped or covalently bound [13,14,15,16,17]. Most of the polymeric micelles, known as Pluronics, consist of triblock PEO–PPO–PEO copolymers: two hydrophilic end-blocks of poly(ethylene oxide) (PEO) and a central hydrophobic block of poly(propylene oxide) (PPO). These copolymers are commercially available in a range of PPO/PEO composition ratios and molecular weights. A relevant number of papers report the application of the Pluronic^®^ surfactants in the preparation of drug delivery systems [18,19,20]. Pluronic smart hydrogel formulations have also been studied for the controlled transport of injectable drugs [13] while other studies have focused on the development of new thermal-sensitive hydrogels for the administration of intranasal vaccines [14]. No paper has reported on their use in protecting drugs from light.

In this work, the use of edible polymer matrices with the addition of antioxidants was investigated as novel photoprotective systems for the preparation of liquid or gel formulations containing the synthesized DHPs. The selected surfactants were Pluronic F-127, which forms micelles consisting of 65 hydrophobic PPO blocks and a corona formed by 200 hydrated PEO blocks, and Pluronic F-108, comprising 50 PPO units and 265 PEO units [15,21]. If the concentration of Pluronics in the aqueous solution exceeds the CMC value, micelles having spherical, cylindrical, lamellar or vesicular morphologies are formed [15].

The study aims to obtain a topical formulation endowed with favorable pharmacokinetic properties to use in the treatment of neuropathic pain that affects a considerable amount of the world population. All the prepared formulations were subjected to photodegradation tests, according to the International Conference on Harmonization (ICH) rules [22]. The photodegradation tests were monitored by spectrophotometry and the kinetic degradation parameters were calculated for all compounds by multivariate curve resolution—alternating least squares (MCR-ALS) [23,24,25,26,27].

## 2. Materials and Methods

### 2.1. Chemicals

HM8 (benzyl 4-(2-hydroxy-3,5-dinitrophenyl)-2,6,6-trimethyl-5-oxo-1,4,5,6,7,8-hexahydroquinoline-3-carboxylate), HM10 (2-(methacryloyloxy)ethyl 4-(3,5-dichloro-2-hydroxyphenyl)-2,6,6-trimethyl-5-oxo-1,4,5,6,7,8-hexahydroquinoline-3-carboxylate) and MD20 (isobutyl 2,6,6-trimethyl-4-(2-hydroxy-3,5-dinitrophenyl)-5-oxo-1,4,5,6,7,8-hexahydroquinoline-3-carboxylate) were synthesized according to modified Hantzsch reactions at the Department of Pharmaceutical Chemistry, Faculty of Pharmacy, Hacettepe University, Ankara, Turkey. Equimolar amounts of 4,4-dimethyl-1,3-cyclohexanedione, 2-hydroxy-3,5-dinitrobenzaldehyde, benzyl (HM8)/isobutyl (MD20) acetoacetate and excess ammonium acetate were dissolved in absolute ethanol and subjected to microwave irradiation (100 W power, constant) for 10 min [6,7]. Upon cooling, the precipitate, obtained by pouring the reaction mixture into ice-water, was filtered and recrystallized from ethanol-water. HM10 was obtained according to the same procedure differently using 3,5-chlorosalicylaldehyde and 2-(methacryloyloxy) ethyl acetoacetate as the aromatic aldehyde and appropriate acetoacetate, respectively [7].

Pluronic^®^ F-127 (MW 12600), F-108 (MW 14600), α-tocopherol and microcrystalline carboxymethylcellulose were purchased from Sigma-Aldrich (Darmstadt, Germany); and ethanol and acetonitrile from J.T. Baker (Deventer, Holland). All chemicals were used without further purification.

### 2.2. Sample Preparation

Due to the almost insolubility of the compounds in water, ten calibration solutions in ethanol were prepared for each compound in concentrations ranging from 5.0 to 30.0 μg mL^−1^. These solutions were used to establish through MCR the mathematical relationships between the concentration of the compounds and their respective spectrophotometric signals. Likewise, three sets (prediction samples) of five one-component solutions were prepared, containing the compounds in the same concentration range used for the calibration sets. These solutions were used to validate the analytical methods defined and to calculate their statistical effectiveness in terms of accuracy and precision.

The micellar solutions were prepared by diluting one compound at a time with Pluronic^®^ surfactant in 10 mL of water. Two of the surfactants tested, namely F-108 and F-127, showed self-aggregation in aqueous solution, forming spherical micelles above a critical micellar concentration (CMC) [15,21]. Therefore, the amount of each surfactant was calculated as an excess of its CMC, measured as 2.30 and 0.43 mol L^−1^ × 10^−3^, respectively, for F-108 and F-127 [21].

A series of micellar solutions was prepared by varying the drug:surfactant concentration ratio according to the values 1:5 and 1:10. These solutions were stirred (200 rpm) for 72 h at room temperature in a glass flask. The micellar size was measured by dynamic light scattering (DLS) using a 90 Plus Particle Size Analyzer (Brookhaven Instruments Corporation, New York, NY, USA) at 25.0 ± 0.1 °C and the incorporation efficiency was calculated by spectrophotometry. Stability tests were also performed on the micellar formulations added with α-tocopherol 5% and stirred at 200 rpm for 10 min.

The micellar-in-gel formulation of each compound was prepared by adding carboxymethylcellulose 0.36 g (gelling agent) to the micellar aqueous solution 10.0 mL, prepared as above described [28,29]. The emulsion was stirred at 200 rpm for 50 min at room temperature to obtain a homogeneous transparent gel.

The photodegradation profiles of these formulations were compared with the standard gel formulations (5 g) containing the free drugs, in absence of the micelles, but in the same drug concentration measured in the micellar formulation. These gels were prepared by emulsifying about 5.0 mg of each compound with 0.36 g carboxymethylcellulose (gelling agent) and 10.0 mL water [28,29].

### 2.3. Photodegradation Study

The photodegradation tests were performed by means of a Suntest CPS+ (Heraeus, Milan, Italy), which uses a Xenon lamp as a light source, providing wavelengths in the range 300–800 nm, according to the ID65 standard source of the ICH rules [22]. The irradiation power was set at 350 W m^−2^, corresponding to 21 kJ m^−2^·min^−1^, at a constant temperature of 25 °C. The experiments were carried out in a dark room to avoid any light interference.

The UV spectra of the ethanol solutions and micellar matrices were recorded in the λ range 200–450 nm by a Perkin-Elmer Lambda 40P Spectrophotometer (Artisan Technology Group, Mercury Drive Champaign, IL, USA) and acquired by UV WinLab^®^ (Perkin-Elmer, Boston, MA, USA).

The analyses were done in triplicate at time 0 and at the sequential time points: 1, 3, 5, 10, 15, 20, 30, 40, 50, 60, 80, 100, 120, 150, 180, 240 and 300 min. The micellar systems were diluted with ethanol, 0.5 to 5 mL.

Gel formulations were irradiated at the following exposure times: 0, 5, 15, 20, 40, 60 and 120 min. The photodegradation tests were performed by uniformly layering 0.5 g of the gel on each of seven glass plates with a layer thickness of 0.25 mm. The gel layering was carried out by placing two 0.25-mm-thick aluminum sheets on the glass at a distance of 2 cm and then layering the gel on the aluminum sheets using a steel spatula. The prepared plates were then exposed to forced irradiation. At various irradiation intervals, each glass plate was sonicated in 25 mL acetonitrile for 10 min at room temperature. Then, 10 mL of the suspension obtained were centrifuged at 5000 rpm for 10 min and the supernatant was analyzed after a 1:10 dilution with ethanol.

### 2.4. MCR Procedure

The MCR algorithm was applied to the spectral data to calculate the drug concentration in the samples using the MATLAB^®^ computer environment software (Mathwork Inc., version 7, Natick, MA, USA). Application of this technique allowed to estimate the number of components, their spectra, the concentration profiles, and the rate constants (k) of the kinetic processes. The MCR methods were elaborated on the standard drug solutions (calibration sets), prepared as above described and validated on external samples with the same concentration range (prediction sets). For each compound, the Limit of Detection (LOD) and Limit of Quantitation (LOQ) were measured.

## 3. Results

### 3.1. DLS Analysis

DLS analysis was performed to determine the mean diameter of the micellar systems. The characteristics of the obtained micelles are reported in Table 1.

**Figure 2:**
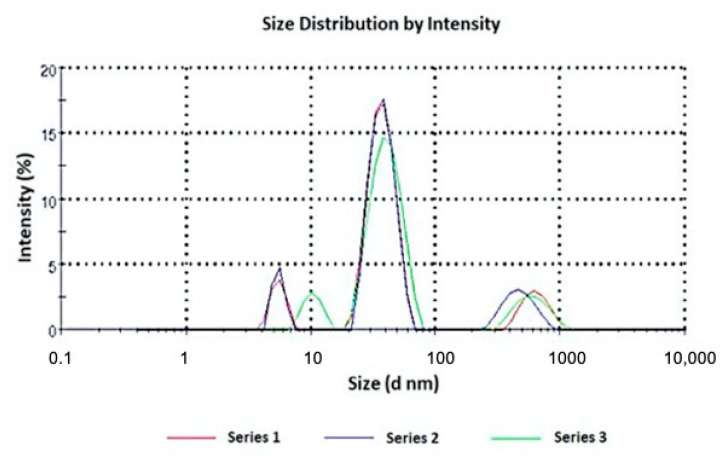
Size distribution of HM10 loaded in F127 micelles, in triplicate.

The micelle size ranged from 22.61 to 54.41 nm and the coexistence of small micelles and large aggregates was also observed (Figure 2). This trend was widely reported in the literature for Pluronic micelles loaded with a hydrophobic drug: an increased hydrophobicity of the micellar core is associated with a higher presence of colloidal aggregates [19,20]. The use of lipophilic substances influences the evolution of the aggregation characteristics of these systems. This effect is attributed to the increased hydrophobicity of the surfactant molecules by reducing the availability of water around them, thus resulting in a rich set of structural transitions in the surfactant aggregates. In fact, hydrophobic drugs are located in the inner core and corona of the micelles, establishing strong interactions with the polymer chains that lead to a decrease in the intermicellar interaction distance and promote the aggregation process. This effect was predominant when HM10 and MD20 were loaded in the F108 and F127 micelles, respectively, for which only large aggregates were recorded.

The graphs representing the size measurement of the other samples are added as Supplementary Materials (Figures S1–S3).

The absence of a chemical interaction between the drugs, the Pluronic surfactant and the carboxymethylcellulose was also demonstrated by FTIR. The typical peaks for each compound were detected in their mixture spectrum, confirming the absence of interactions. For example, the overlapping of the FTIR spectra of HM10, Pluronic F127, carboxymethylcellulose and their mixture is shown in the Supplementary Material (Figure S4).

A physical-chemical characterization was also performed on the micellar formulations to which 5% α-tocopherol were added, and the obtained data are reported in Table 2.

### 3.2. MCR Processing

Absorbance spectra of the ethanol calibration samples were recorded over the wavelength range 200–450 nm. A first selection of the most useful wavelengths was performed, discarding the wavelengths below 210 nm, which are usually affected by high variability or instrumental noise that can make the model unstable, as well as the wavelengths over 390 nm, due to the absence of signals. Therefore, the MCR elaboration was applied on the spectral data between 210 and 390 nm.

The values of the lack of fit (%LOF), which is an index of the fit quality of the MCR results, was less than 4.5% in all experiments. LOD values calculated were 1.2, 0.99 and 1.1 μg/mL for HM8, HM10 and MD20, respectively. LOQ was in the range 2.4–45.1 μg/mL for HM8, 2.1–46.5 μg/mL for HM10 and 2.5–44.5 μg/mL for MD20.

### 3.3. Photodegradation of the Ethanol Solutions

**Figure 3:**
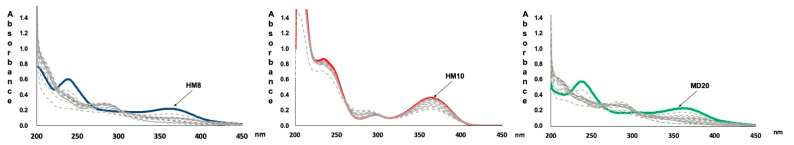
UV spectra recorded along the photodegradation test on the ethanol solutions of HM8, HM10 and MD20.

Photostability of the DHP compounds was tested by exposing to light the ethanol one-component solutions at a concentration of 20.0 μg mL^−1^. These solutions were placed in quartz cuvettes and subjected to a photodegradation test as above described. Figure 3 shows the sequences of the absorbance spectra in the range 200–450 nm recorded along the photodegradation tests of HM8, HM10 and MD20 at zero time and at several intervals up to 5 h.

These data were processed by MCR to estimate the number of photoproducts, as well as their spectra and concentration profiles. The photodegradation rate constant (*k*) was calculated for these experiments, showing in all cases first-order kinetics. The following equation describe the degradation process:

ln[% DHP] = −*k* × *t* + 4.67

where % DHP is the percentage concentration of the residual drug; *t* is the time, expressed in minutes; and 4.67 is the natural logarithm of the starting concentration percentage (100%).

Considering that a pharmaceutical formulation whose quantity of active ingredient falls below 90% of the initial value can no longer be used, the parameter *t*_0.1_ (time to obtain a 10% degradation) was selected to compare the behavior of the prepared formulations when exposed to light. In addition, the shelf-life of the formulations was also compared by using the parameter *t*_0.5_ (time to obtain a 50% degradation). The calculated kinetic parameters collected in all the photodegradation experiments are listed in Table 3. The data were the average of three experiments with the values of the relative standard deviation (RSD) for all parameters within the range 1.66–5.04%.

**Figure 4:**
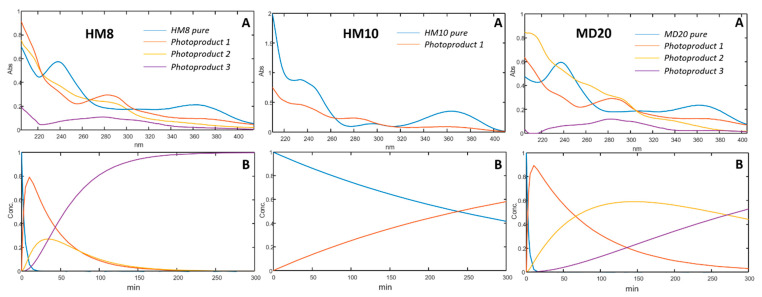
Spectra (**A**) and concentration profiles (**B**) for HM8, HM10 and MD20, and their photoproducts.

The graphs elaborated from the MCR analysis are shown in Figure 4. For each compound, the absorbance spectra and the concentration profiles of the drugs and relative photoproducts are shown. As reported in our previous works, the oxidation of the dihydropyridine ring to the pyridine-based photoproduct was confirmed for all the tested compounds. Furthermore, the formation of secondary photoproducts for HM8 and MD20 was detected, based on the presence of nitro-groups on the phenyl ring, which accelerates the oxidation process due to the delocalization of the negative charge [30,31].

### 3.4. Photodegradation of Micellar Solutions

Preparation of the micellar solutions containing the studied compounds was optimized by performing various experiments to define the drug:surfactant ratio and the amount of the antioxidant. In this way, an increase in the solubility for the compounds in the micellar solution was obtained.

**Figure 5:**
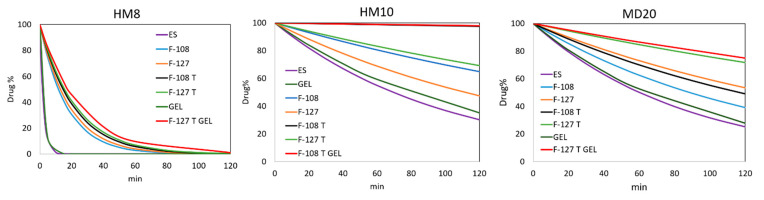
Photodegradation of HM8, HM10 and MD20 in ethanol, surfactant solutions and gel formulations. ES, ethanol solution; T, α-tocopherol.

For each compound, the micellar solutions showing an entrapping percentage higher than 20% (Table 1) were selected to be subjected to the photodegradation test. To these formulations were added 5% α-tocopherol just before the photodegradation test. Figure 5 shows the photodegradation profiles of HM8, HM10 and MD20 in the micellar solutions, compared with those carried out from the ethanol solutions. The kinetic parameters, listed in Table 3, were calculated by applying MCR analysis to the spectral data. These formulations also followed first-order kinetics for the degradation process.

### 3.5. Photodegradation of the Gel Formulations

**Figure 6:**
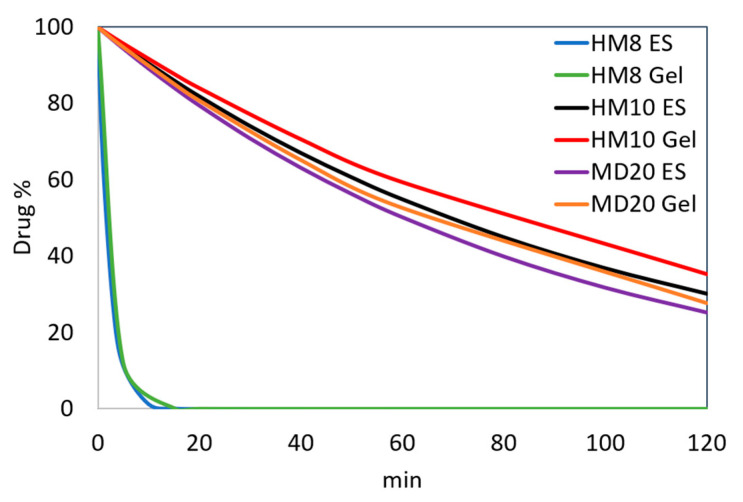
Comparison of the photodegradation profiles of HM8, HM10 and MD20 in ethanol and gel formulations.

The stability of the micellar solutions was also tested in the gel formulation. The micellar systems of the three compounds that showed the best performance, in terms of entrapping percentage and photostability, were formulated in gel, as above described. The photodegradation profiles of these formulations were compared with those of a standard gel prepared in the same conditions and containing the free drugs. Figure 6 compares the photodegradation rates on these samples.

The kinetic parameters calculated by MCR are listed in Table 3. Figure 5 shows the photodegradation profiles of HM8, HM10 and MD20 in a micellar-gel formulation compared with those of the free compounds in the gel. First-order degradation kinetics were confirmed also for these formulations.

## 4. Discussion

This paper presents the photodegradation study of newly synthesized DHP-based hexahydroquinolines (HM8, HM10 and MD20) with T-type calcium channel blocking activity (Cav3.2), which is considered a new approach to develop novel analgesics, particularly against neuropathic pain. We formulated them in liquid and gel preparations with the aim of minimizing their sensitivity to light. Photostability of the compounds was first tested in ethanol and gel under standard conditions. The %LOF values, resulting below 4.5% in all the experiments, confirmed the quality and robustness of the MCR data processing from the photodegradation tests.

The sequence of the spectra collected during the photodegradation experiments on the ethanol solutions (Figure 3) showed a decrease of the peak in the 350–390 nm zone for all the compounds, typical of the dihydropyridine structure, and the simultaneous increase of a new peak in the 260–310 nm region. According to our previous studies [30,31], the different chemical groups on the phenyl and dihydropyridine rings lead to different kinetic photodegradation profiles. The presence of chlorine atoms on the phenyl ring of HM10 confers greater stability than the nitro groups in HM8 and MD20. MCR elaboration of the spectral data confirmed this trend, assuming the formation of only the pyridine derivative for HM10 (Figure 4A). Two photoproducts were instead detected during degradation of both HM8 and MD20, corresponding to the pyridine derivatives and to the subsequent reduction of the nitro groups in Positions 3 and 5 on the phenyl ring to nitroso groups. Traces of a third by-product were observed after prolonged exposure to light, but devoid of any characterizing signal. This photodegradation profile was carried out also when the drugs were formulated in gel. As compared in Figure 6, HM8 showed a very fast degradation process in both matrices, with an almost complete disappearance after 10 min of light exposure. In contrast, HM10 and MD20 showed greater stability, with a *t*_0.1_ value of 10.54 and 9.16 min in ethanol and 12.11 and 9.87 min in gel, respectively.

Due to the marked photosensitivity of the studied molecules, the increase in their photostability was investigated by incorporating the compounds into polymeric micelles.

At first, the use of the Pluronic F-68 surfactant, comprising 29 PPO units and 153 PEO units [15], was tested. Unfortunately, the use of F-68 did not produce satisfactory results, because the entrapping percentage was below 10% in all the experiments, despite several modifications of the experimental conditions. Indeed, difficulties in using Pluronic F-68 in drug formulation have been reported, due to its high CMC, resulting in a low drug load and poor dilution stability [32]. For these reasons, the micellar solutions prepared with this surfactant were eliminated from the subsequent study. The aim of the work was achieved when the compounds were entrapped in micelles formed by using Pluronics F-108 and F-127, showing entrapping percentages above 20% in all formulations. The best results, with values in the range 59–69%, were obtained for HM8 and MD20 entrapped in Pluronic F-127 micelles, whose dimensions were much larger than those formed using Pluronic F-108. HM10, which has a higher molecular weight, formed larger micelles when entrapped in Pluronic F-108. The incorporation percentage of the DHP compounds into the two selected Pluronic surfactants was carried out by spectrophotometry and the values are listed in Table 1.

The micellar solutions prepared with F-108 and F-127 were subjected to photodegradation and the spectra recorded along these experiments were processed by MCR. A marked increase in the light stability was observed for HM10 in both the micelle matrices, showing *t*_0.1_ values of 29.27 and 16.97 min when using F-108 and F-127, respectively. The formation of the pyridine by-product alone was detected. An almost complete photostability was observed for this compound when 5% α-tocopherol was added to the micellar solutions. As reported in Table 2, α-tocopherol loaded in the micellar systems led to a significant increase in the micelle size. The percentage entrapment of the drugs was slightly decreased and the antioxidant was entrapped at an average of 36%. This behavior did not affect the Z-potential values.

This result demonstrated the importance of the presence of an antioxidant in opposing the photodegradation process of DHP compounds, which mainly involves the oxidation process of the dihydropyridine ring. A clear decrease in the photodegradation rate was shown also for MD20, confirming the validity of the inclusion process in the polymeric micelles. The best results were obtained when this compound was entrapped in the F-127 micelles, showing a *t*_0.1_ value of 20.18 min. This formulation was analogously added with α-tocopherol, providing a further increasing of light stability, with a *t*_0.1_ value of 38.17 min. In contrast, the results from the tests on HM8 were not satisfactory, despite the micellar formulations showing a significant increase in light stability, showing a *t*_0.1_ value of 1.97 when entrapped in the F-127 micelles and *t*_0.1_ value of 2.37 min when α-tocopherol was added. The overall *t*_0.1_ values are summarized in Table 3.

The most stable micellar sample for each product was finally formulated in a gel matrix and subjected to photodegradation. The experimental procedure, applied in the preparation of the gels, involved the use of plate glasses to be exposed in the irradiation chamber. The use of only seven plates was due to the size of this chamber. Considering that the *t*_0.1_ value for most of the prepared formulations were less than 45 min, we used shorter irradiation times for the gels than in the experiments performed on the drugs in solution. As shown in Figure 5, the HM8 compound in the micelle-in-gel showed a ten-fold increase in the *t*_0.1_ value (2.69 min) compared to the standard gel but the photodegradation profile of the micelles-in-gel formulation was like that of the micellar solution. This result was not considered satisfactory for making a light-stable topical formulation. For this compound, the advantage in using the micellar system was limited to improve the water solubility.

Satisfactory results were obtained for MD20, which showed a remarkable increase in light stability, with a *t*_0.1_ value of 43.90 min. In addition, the light stability was constant for long exposure times, recording a residual concentration of 50% after 288 min. The best result was achieved for the HM10 compound in micelles-in-gel, which maintained a drug concentration of 90% for more than 8 h under stressing light. For these two compounds, UV light likely induced modifications on the polymers and therefore the light exposure of the incorporated drugs was limited. The polymer envelopes were able to isolate the drugs from the surrounding environment and, thus, the UV irradiation did not penetrate below the first molecular layers.

The proposed micelle-in-gel matrices were demonstrated to be able to both entrap water-insoluble drugs and assure high photostability. Furthermore, the polymeric micelles have a high drug-loading capacity and good dispersion characteristics in the body. Based on these results, the study presents some limitations. The proposed formulations for HM8, despite the improved stability compared to the ethanolic solution, showed complete degradation after a short exposure time to light. In addition, our topical gel formulations need to be examined for their analgesic effects to be potential candidates in the treatment of neuropathic pain through blocking T-type calcium channels.

## 5. Conclusions

A novel approach is proposed for the development of light-stable liquid formulations containing newly synthesized Dihydropyridines with T-type calcium channel blocking activity. Satisfactory results were achieved when two of these compounds were incorporated into micelles formed with Pluronics F-127 and F-108 as surfactants and α-tocopherol as antioxidant. The compounds HM10 and MD20 in micelles-in-gel showed a drug concentration of 90% after 8 and 1 h of light exposure. Furthermore, the use of surfactants greatly favored the solubility in water of all the tested DHP molecules. The proposed matrices demonstrated a remarkable ability in protecting the studied DHPs from light to develop topical formulations. As T-type calcium blockers hold therapeutic value for pain intervention, these formulations can be potential candidates in pain therapy after their analgesic effects are further investigated.

## Figures and Tables

**Table 1:** Characteristics of the Pluronic micellar solutions.

Compound	Surfactant	Drug: Surfactant Ratio	Micellar Size (nm ± DS)	PDI	Z Potential (mV)	Drug Entrapping %
HM8	F-108	1:10	22.61 ± 0.11	0.218	−12.7 ± 2.44	29.1
	F-127	1:10	54.41 ± 0.63	0.263	−9.89 ± 1.89	69.2
HM10	F-108	1:5	Aggregates	-	−4.78 ± 3.23	20.3
	F-127	1:5	34.75 ± 1.13	0.389	−8.76 ± 2.23	20.9
MD20	F-108	1:10	23.74 ± 2.25	0.382	−9.14 ± 0.08	28.2
	F-127	1:10	Aggregates	-	−4.56 ± 1.25	58.9

**Table 2:** Characteristics of the Pluronic micellar solutions loaded with 5% α-tocopherol.

Compound	Surfactant	Z-Potential (mV)	Micellar Size (nm ± DS)	PDI	Drug Entrapping %	α-Tocopherol Entrapping %
HM8	F-108	−5.99 ± 1.05	98.02 ± 5.45	0.521	25.3	31.1
	F-127	−6.89 ± 1.22	157.9 ± 15.19	0.851	61.1	33.9
HM10	F-108	−7.78 ± 1.93	Aggregates	-	18.3	37.6
	F-127	−6.76 ± 2.94	126.0 ± 13.76	0.762	19.1	39.8
MD20	F-108	−7.92 ± 1.76	175.1 ± 8.09	0.382	25.4	35.4
	F-127	−8.76 ± 0.95	Aggregates	-	54.6	37.8

**Table 3:** Degradation kinetic parameters calculated for the HM8, HM10 and MD20 formulations.

Compound	Formulation	*k* (×10^−3^)	*t*_0.1_ (min)	*t*_0.5_ (min)	*R* ^2^
HM8	Ethanol solution	7.28	0.24	1.59	0.994
	F-108	0.99	1.78	11.71	0.992
	F-127	0.89	1.97	12.96	0.934
	F-108-tocopherol	0.79	2.23	14.65	0.988
	F-127-tocopherol	0.74	2.37	15.61	0.995
	Standard gel	7.04	0.25	1.64	0.912
	F-127-tocopherol gel	0.65	2.69	17.73	0.941
HM10	Ethanol solution	0.17	10.54	69.31	0.974
	F-108	0.06	29.27	192.54	0.957
	F-127	0.10	16.97	111.62	0.940
	F-108-tocopherol	0.00	501.72	-	0.909
	F-127-tocopherol	0.05	34.43	226.52	0.976
	Standard gel	0.15	12.11	79.67	0.996
	F-108-tocopherol gel	0.00	605.52	-	0.975
MD20	Ethanol solution	0.19	9.16	60.27	0.985
	F-108	0.13	13.51	88.87	0.976
	F-127	0.09	20.18	132.79	0.997
	F-108-tocopherol	0.10	17.74	116.69	0.965
	F-127-tocopherol	0.05	38.17	251.14	0.901
	Standard gel	0.18	9.87	64.90	0.955
	F-127-tocopherol gel	0.04	43.90	288.81	0.943

## Data Availability

All data presented in this study are included within the article.

## Supplementary Materials

The following are available online at https://www.mdpi.com/article/10.3390/pharmaceutics13040527/s1. Figure S1. Size distribution of a triplicate of HM8 loaded in F108 micelles, Figure S2. Size distribution of a triplicate of HM8 loaded in F127 micelles, Figure S3. Size distribution of a triplicate of MD20 loaded in F108 micelles, Figure S4. Overlapping of FTIR spectra of HM10, Pluronic F127, carboxymethylcellulose and their physical mixture prepared in the concentration ratio drug/surfactant/gelling agent used in the micellar formulations.
