# Supplementary material for: Use of Pluronic Surfactants in Gel Formulations of Photosensitive 1,4-Dihydropyridine Derivatives: A Potential Approach in the Treatment of Neuropathic Pain
Source: Pharmaceutics. 2021 Apr 10;13(4):527. doi: 10.3390/pharmaceutics13040527 (PMC8070562; doi:10.3390/pharmaceutics13040527)
Supplement: Supplementary file 1 [file pharmaceutics-13-00527-s001.pdf]

pharmaceutics

MDPI

Supplementary Materials: Use of Pluronic Surfactants in Gel Formulations of Photosensitive 1,4-Dihydropyridine Derivatives: A Potential Approach in the Treatment of Neuropathic Pain

Giuseppina Ioele, Rita Muzzalupo, Miyase Gözde Gündüz, Michele De Luca, Elisabetta Mazzotta, Fedora Grande, Maria Antonietta Occhiuzzi, Antonio Garofalo and Gaetano Ragno

Supplementary Material

Size distribution of each sample

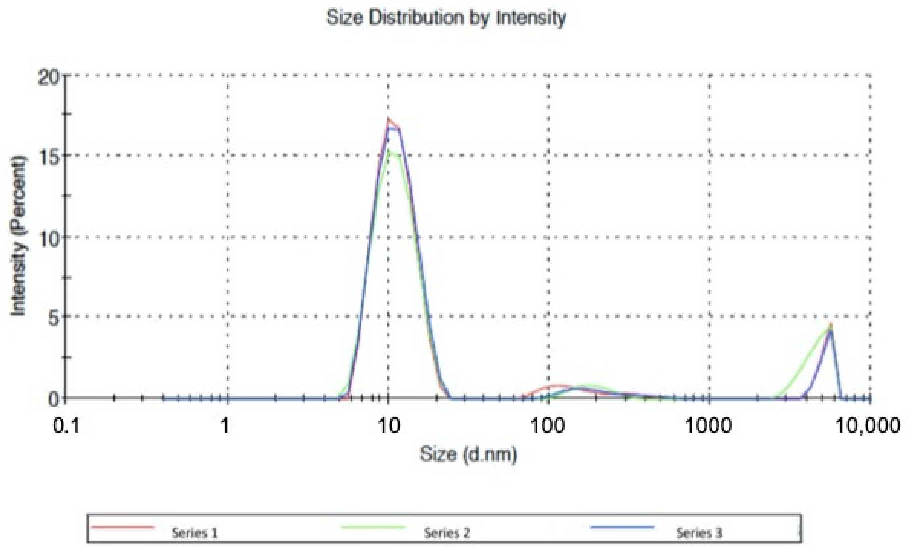

Figure S1. Size distribution of a triplicate of HM8 loaded in F108 micelles.

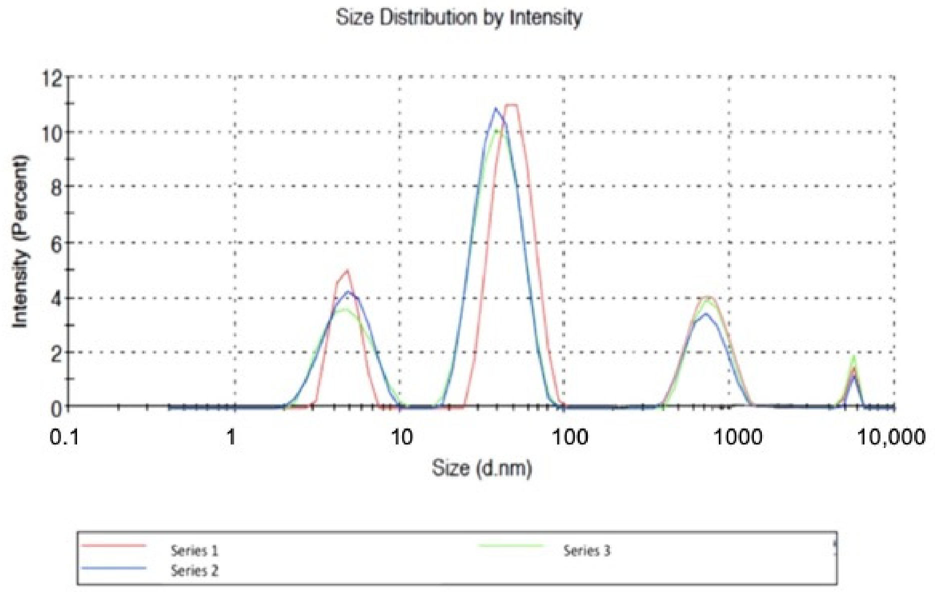

Figure S2. Size distribution of a triplicate of HM8 loaded in F127 micelles.

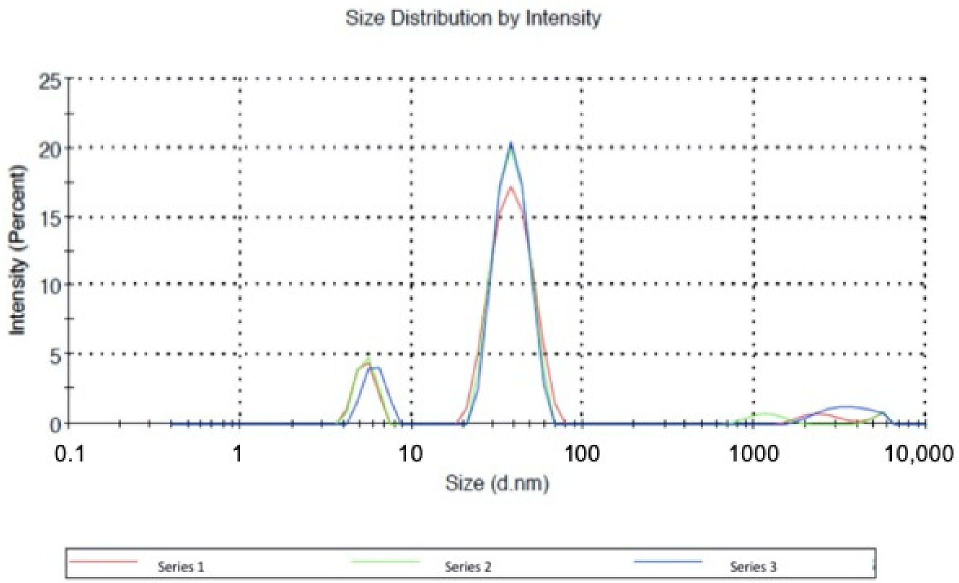

Figure S3. Size distribution of a triplicate of MD20 loaded in F108 micelles.

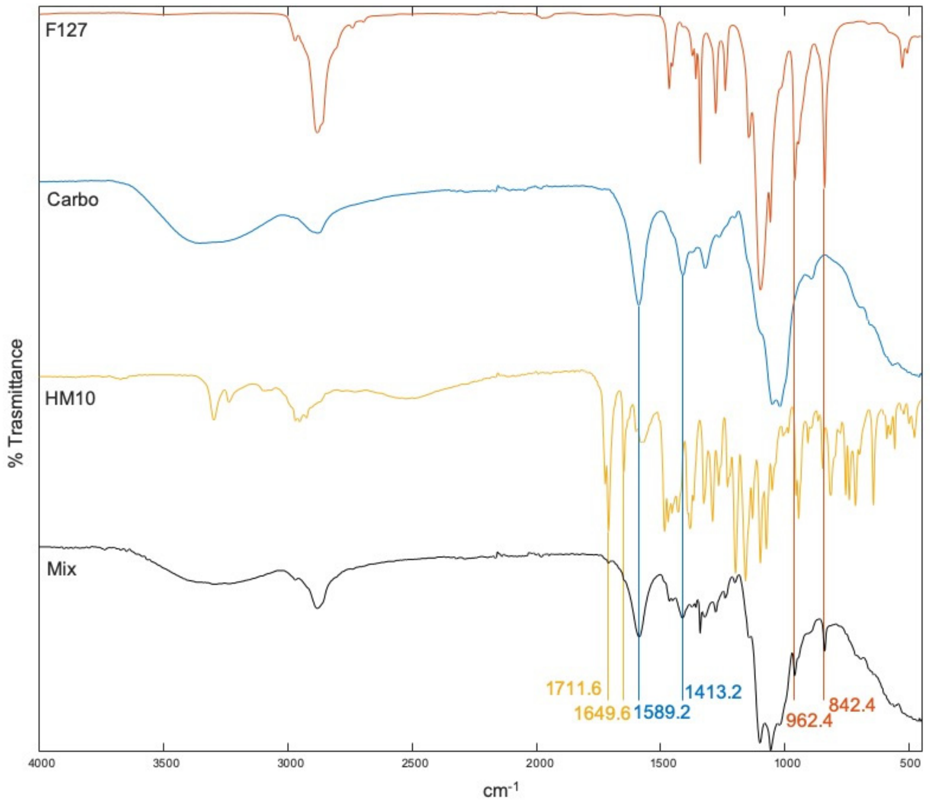

Figure S4. Overlapping of FTIR spectra of HM10, Pluronic F127, carboxymethylcellulose and their physical mixture prepared in the concentration ratio drug/surfactant/gelling agent used in the micellar formulations. Major peaks typical for each compounds and present in the mixture are marked. There is no significant change in the absorption spectra for the drug when prepared in mixture.
